# Suilysin-induced Platelet-Neutrophil Complexes Formation is Triggered by Pore Formation-dependent Calcium Influx

**DOI:** 10.1038/srep36787

**Published:** 2016-11-10

**Authors:** Shengwei Zhang, Yuling Zheng, Shaolong Chen, Shujing Huang, Keke Liu, Qingyu Lv, Yongqiang Jiang, Yuan Yuan

**Affiliations:** 1 State key Laboratory of Pathogen and Biosecurity, Beijing Institute of Microbiology and Epidemiology, Beijing 100071, China; 2 Department of clinical laboratory, Dongfang Hospital, Beijing University of Chinese Medicine, Beijing 100078, China; 3 Department of clinical laboratory, Beijing You’an hospital, Capital Medical University, Beijing 100069, China

## Abstract

Platelet activation and platelet–neutrophil interactions have been found to be involved in inflammation, organ failure and soft-tissue necrosis in bacterial infections. *Streptococcus suis*, an emerging human pathogen, can cause streptococcal toxic-shock syndrome (STSS) similarly to *Streptococcus pyogenes*. Currently, *S. suis*–platelet interactions are poorly understood. Here, we found that suilysin (SLY), the *S. suis* cholesterol-dependent cytolysin (CDC), was the sole stimulus of *S. suis* that induced platelet-neutrophil complexes (PNC) formation. Furthermore, P-selectin released in α-granules mediated PNC formation. This process was triggered by the SLY-induced pore forming-dependent Ca^2+^ influx. Moreover, we demonstrated that the Ca^2+^ influx triggered an MLCK-dependent pathway playing critical roles in P-selectin activation and PNC formation, however, PLC-β-IP3/DAG-MLCK and Rho-ROCK-MLCK signalling were not involved. Additionally, the “outside-in” signalling had a smaller effect on the SLY-induced P-selectin release and PNC formation. Interestingly, other CDCs including pneumolysin and streptolysin O have also been found to induce PNC formation in a pore forming-dependent Ca^2+^ influx manner. It is possible that the bacterial CDC-mediated PNC formation is a similar response mechanism used by a wide range of bacteria. These findings may provide useful insight for discovering potential therapeutic targets for *S. suis*-associated STSS.

*Streptococcus suis* serotype 2 (SS2) infection is one of the major causes of septicaemia and meningitis in pigs and humans^1^,^2^. In 2005, China reported more than 200 human cases with an unusual clinical presentation of streptococcal toxic-shock syndrome (STSS) and a mortality rate of up to 20%^3^. Thrombocytopenia and multisystem dysfunction, such as liver failure and heart failure, were found in more than half of the STSS patients. Moreover, purpura and gangrenous necrosis were the typical skin manifestations in STSS patients^4^,^5^.

Platelet–neutrophil interactions have been found to be involved in inflammation^6^, organ failure^7^ and soft-tissue necrosis^8^,^9^ in some life-threatening infectious diseases by direct injury to the endothelium. The myonecrosis tissue specimen from a patient with *S. pyogenes*-associated STSS showed numerous platelet/leukocyte thrombi, and streptolysin O (SLO) of *S. pyogenes* mediated the platelet-neutrophil complexes (PNC) formation in a P-selectin-dependent manner^9^. The α-hemolysin of community-associated methicillin-resistant *S. aureus* (CA-MRSA) has been reported to induce PNC formation via platelet P-selectin, suggesting that *S. aureus* α-hemolysin may contribute to alveolar capillary leakage, haemorrhage and lung destruction during MRSA necrotizing pneumonia^10^. However, the molecular signalling pathways induced by these bacterial toxins to activate platelet P-selectin are mostly unknown. Moreover, the interactions between platelets and *S. suis* and the molecular mechanism underlying the *S. suis*-induced platelet-neutrophil interactions remain poorly understood.

P-selectin is a member of the selectin family of cell surface receptors. Platelet P-selectin primarily binds P-selectin glycoprotein ligand-1 (PSGL-1) on neutrophils, mediates the platelet–neutrophil interactions^11^,^12^,^13^, is stored in α-granules of platelets and is rapidly secreted to the surface upon activation by agonists [e.g., ADP, platelet-activating factor (PAF)]^14^. The PLC-β-IP3/DAG-MLCK^14^,^15^,^16^ and Rho-ROCK-MLCK signalling pathways^17^ usually mediate the agonist-induced platelet activation. The PLC-β-IP3/DAG-MLCK signalling is often accompanied by an increase in intracellular Ca^2+^, which binds to calmodulin^18^. The final signalling events in the above mentioned pathway are phosphorylation of MLC by MLCK that leads to actin–myosin interactions, consequently resulting in platelet degranulation^14^. Additionally, during platelet activation, the agonists induce the “inside-out” signalling, in turn the ligand-occupied GPIIb/IIIa receptor usually reinforces activation of the “outside-in” signalling, leading to secretion of α-granules^19^.

Suilysin (SLY) is a 497 amino-acid protein belonging to the cholesterol-dependent cytolysin (CDC) family, which has more than 20 members, including pneumolysin (PLY) and SLO, expressed by *S. pneumoniae* and *S. pyogenes*, respectively. Like other members of the CDC family produced by Gram-positive bacteria, a classical feature of these toxins is their ability to create transmembrane pores in cholesterol-containing membranes, thereby causing cell lysis^20^,^21^. In this study, SLY was the sole stimulus responsible for PNC formation induced by *S. suis*. We also found that the SLY-induced pore forming-dependent Ca^2+^ influx triggered human platelet P-selectin release to the plasma membrane, where it mediated adhesion of platelets to neutrophils. Moreover, the Ca^2+^-MLCK-dependent signalling was required for a significant release of P-selectin, which was essential for SLY to induce PNC formation. Our findings showed that PLY and SLO-induced PNC formation was also triggered by pore formation-dependent Ca^2+^ influx, and suggest a possible similar mechanism to SLY. These findings may provide useful insight for discovering potential therapeutic targets for *Streptococci*-associated STSS.

## Results

### SLY is the sole stimulus responsible for PNC formation induced by *S. suis*

**Figure 1:**
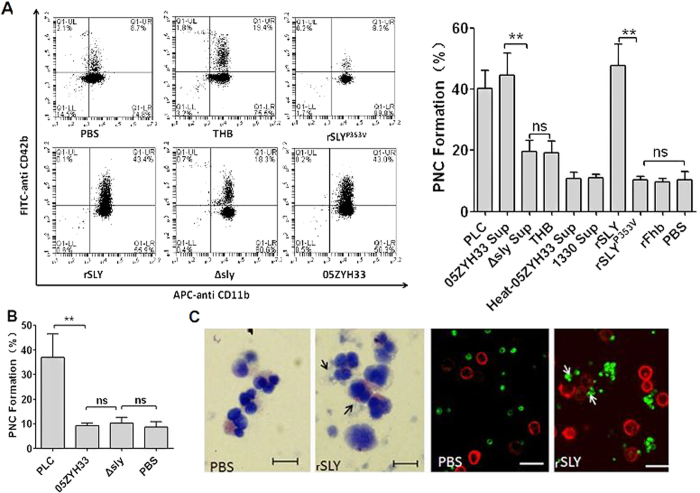
SLY plays important roles in PNC formation induced by *S. suis*. Blood samples (100 μL) were preincubated with APC-CD11b and FITC–CD42b, and then stimulated with 10 μL of (**A**) bacteria-free stationary phase culture supernatant from *S. suis*, recombinant proteins (1 μg/mL), PLC and THB/PBS, (**B**) the washed bacteria. PNC formation in human whole blood was quantitated by dual-colour flow cytometry. The histograms shown in A (left panel) are from one representative experiment. Data in A (right panel) are expressed as the mean ± SD of three independent experiments, with each experiment using blood from a different donor. **P < 0.01; ns, no significance. *Clostridium perfringens* phospholipase C (PLC) served as a positive control; THB/PBS is the negative control for culture supernatant or proteins; 05ZYH33, wild type strain; ∆sly, the isogenic sly mutants; 1330, SLY-negative Canadian strain; Heat-05ZYH33, heat-inactive 05ZYH33; Sup, supernatant; rSLY, recombinant SLY; rSLY^P353V^, recombinant non-hemolytic mutant of SLY^P353V^; rFhb, recombinant factor H-binding protein, an irrelevant protein that was purified by the same procedure as SLY. (**C**) rSLY-induced PNC was observed in whole blood samples by Wright’s stain or by confocal laser scanning microscopy (PMNs were marked in red by APC-CD11b and platelets were marked in green by FITC–CD42b). Black and white arrows point to PNC. The bar represents 10 μm.

To elucidate the stimulus inducing PNC formation, bacterial cells and stationary phase culture supernatants from *S. suis* strains were tested for PNC formation activity in human blood. 05ZYH33 supernatant significantly induced PNC formation significantly (*P* < 0.01, Fig. 1A), whereas the bacterial cells did not (Fig. 1B), suggesting that there are secreted stimuli that induce PNC formation. SLY is an important secreted toxin of *S. suis*; high levels are produced at the end of the exponential growth phase^22^; therefore, we used it for testing PNC-inducing activity. Interestingly, neither Δsly bacterial cells nor the supernatant induced PNC formation as background control Todd-Hewitt broth (THB)/PBS (Fig. 1). Moreover, the supernatant of the SLY-negative Canadian strain 1330 culture and heat-inactivated 05ZYH33 supernatant failed to induce PNC formation (Fig. 1A). Taken together, these results indicate that SLY was the sole stimulus of *S. suis* involved in this process. To further elucidate the mechanism of SLY-stimulated PNC formation, recombinant SLY (rSLY) was generated and it induced PNC formation, but its non-hemolytic mutant, SLY^P353V^, and the irrelevant control protein recombinant factor H-binding protein (rFhb) did not (Fig. 1A). Finally, SLY-induced PNC formation was detected by Wright’s staining of blood smears and immunofluorescence confocal microscopy (Fig. 1C). These data imply that SLY was the sole *S. suis* protein that stimulated PNC formation in human whole blood.

### SLY-induced PNC formation is mediated by P-selectin on human platelets

**Figure 2:**
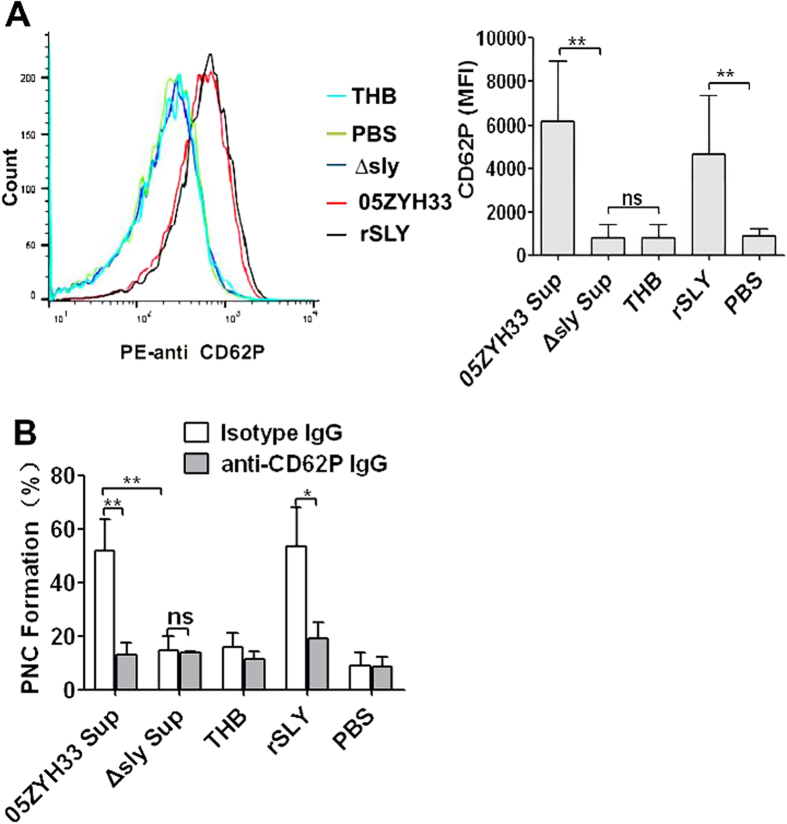
P-selectin mediates the SLY-induced PNC formation. (**A**) *S. suis* culture supernatant or rSLY protein (1 μg/mL)-induced CD62P release from platelets in human blood was assessed by flow cytometry. Representative histograms of the MFI of CD62P are shown in the left panel. (**B**) SLY-induced PNC formation in human blood was assessed in the presence of anti-CD62P blocking antibody (10 μg/mL) or an isotype-matched control antibody (10 μg/mL). THB and PBS are the negative controls for culture supernatant and proteins, respectively. Data in (**A**) right panel and (**B**) are given as the mean ± SD of three independent experiments from three different blood donors. **P < 0.01; ns, no significance; 05ZYH33, wild type strain; ∆sly, isogenic sly mutants; Sup, supernatant; rSLY, recombinant SLY.

During platelet activation, agonists induce platelet shape changes and granular secretion^14^. P-selectin is usually found in the membrane of the platelet secretory granules (α-granules) and is often used as a measure of α-granules release. Here, 05ZYH33 supernatant and rSLY significantly induced P-selectin release to the platelets’ surface (CD62P, *P* < 0.001; Fig. 2A), but the supernatant of ∆sly and the THB/PBS controls did not. These results indicate that SLY was the sole protein of *S. suis* that activated human platelets and induced α-granules release.

P-selectin is a member of the selectin family of cell surface receptors, which primarily mediates neutrophil–platelet, platelet–platelet and monocyte–platelet interactions^14^. Therefore, a specific monoclonal antibody for P-selectin was used to explore the role of P-selectin in PNC formation induced by SLY. Pre-incubation of human blood with a functional neutralizing monoclonal antibody (clone AK-4) abrogated 05ZYH33 supernatant- and rSLY-induced PNC formation to the level of the ∆sly supernatant or the background THB/PBS level (Fig. 2B), indicating that P-selectin principally mediated PNC formation induced by SLY.

### SLY-induced platelet P-selectin release and PNC formation depend on pore formation in platelets

**Figure 3:**
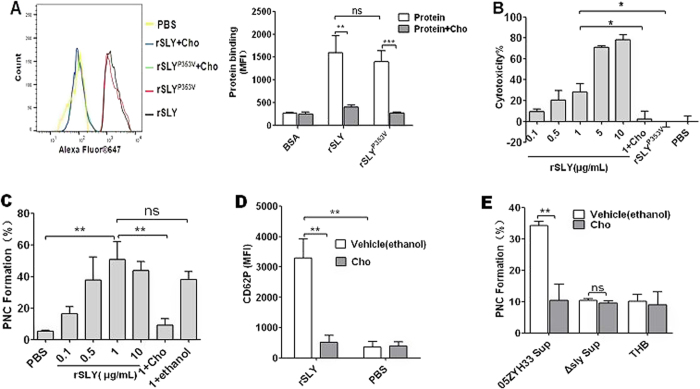
SLY-induced PNC formation depends on pore formation in platelets. (**A**) The binding of rSLY/rSLY^P353V^ (1 μg/mL) to platelets and the cholesterol inhibiting effect was determined by flow cytometry. Representative histograms of the MFI of proteins binding are shown in the left panel. Data in right panel are given as the mean ± SD of three independent experiments from three different blood donors. (**B**) The cytotoxicity of rSLY to platelets and the cholesterol inhibiting effect were assessed by an LDH assay (Methods section). Unpaired two-tailed Student’s t test was used for statistical analysis. (**C**) Dose response of rSLY-induced PNC formation and the cholesterol inhibiting effect. (**D**) The cholesterol inhibiting effect on SLY-induced CD62P release in human blood was assessed by flow cytometry. rSLY (1 μg/mL) and cholesterol (100 μg/mL) were used. (**E**) The cholesterol (100 μg/mL) effect on *S. suis* supernatant-induced PNC formation was detected by flow cytometry. THB and PBS are the negative controls for culture supernatant and proteins, respectively. Cholesterol was dissolved in ethanol. rSLY, recombinant SLY; Cho, cholesterol; 1 + Cho, 1 μg/mL rSLY added to cholesterol. Data in (**A–E**) are given as the mean ± SD of three independent experiments from three different blood donors. **P < 0.01; ns, no significance; 05ZYH33, wild type strain; ∆sly, isogenic sly mutants; Sup, supernatant.

CDCs can bind to membrane cholesterol, to create large pores (350–450 Å in diameter) and consequently lyse the target cells^23^,^24^. Free cholesterol can inhibit CDC-induced pore formation^20^,^21^. To determine whether SLY-induced PNC formation is due to pore formation, the cholesterol inhibition assay was used. As shown in Fig. 3A, rSLY could bind to human platelets. As expected, this binding was inhibited by cholesterol. In further study, rSLY exhibited strong cytotoxicity when its concentration is no less than 5 μg/mL (Fig. 3B). Substantial PNC formation occurred when rSLY was 1 μg/mL (Fig. 3C) and the cytotoxicity was ~28% at this concentration (Fig. 3B). Cholesterol (100 μg/mL) that inhibited SLY-induced cytotoxicity (Fig. 3B) also abrogated the SLY-induced PNC formation (Fig. 3C). Although the non-hemolytic recombinant mutant, rSLY^P353V^ also could bind to platelets (Fig. 3A), it lost cytotoxicity to platelets (Fig. 3B), which therefore failed to induce PNC formation (Fig. 1A). Furthermore, 1 μg/mL rSLY-induced P-selectin release was also significantly reduced by cholesterol (100 μg/mL) to the background level (Fig. 3D). More importantly, cholesterol fully inhibited 05ZYH33 supernatant-induced PNC formation (Fig. 3E). These results indicate that SLY-induced P-selectin release and PNC formation were pore dependent.

### SLY-induced platelet P-selectin release and PNC formation requires pore formation-dependent Ca^2+^ influx

**Figure 4:**
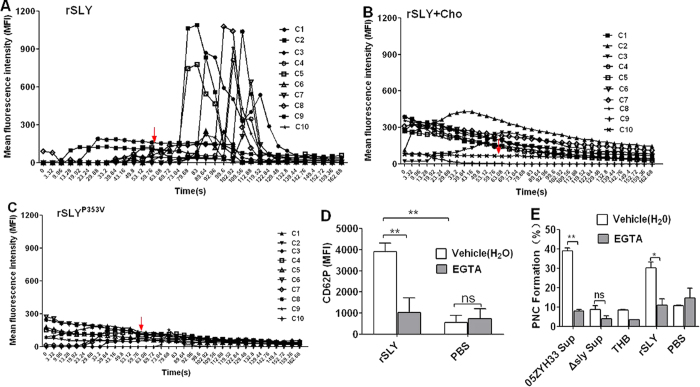
SLY-induced pore formation-dependent Ca^2+^ influx triggers PNC formation. (**A–C**) rSLY induces Ca^2+^ influx in human platelets. The purified platelets marked with Fluo-8 were resuspended in HBSS (with 2 mM Ca^2+^) and rSLY/rSLY^P353V^ (1 μg/mL) or rSLY that was pretreated by cholesterol (10 μg/mL). The Ca^2+^ influx in platelets was observed using an FV1000 confocal laser scanning microscope. The Ca^2+^ influx in platelets was observed using an FV1000 confocal laser scanning microscope. The following mean fluorescence intensity (MFI) of Ca^2+^ mobilization was recorded by Series Analysis (XY-T) software in FV1000. C1-C10, cell 1-cell 10. The video of these results were displayed in Supplementary Information. (**D**) The EGTA effect on rSLY-induced CD62P release from platelets in human blood was assessed by flow cytometry. (**E**) The EGTA (3 mM) effect on *S. suis* supernatant-induced PNC formation was detected by flow cytometry. THB and PBS are the negative controls for culture supernatant and proteins, respectively. EGTA was dissolved in H_2_O. Data in B and C are given as the mean ± SD of three independent experiments from three different blood donors. **P < 0.01; ns, no significance; Cho, cholesterol; rSLY, recombinant SLY; 05ZYH33, wild type strain; ∆sly, isogenic sly mutants; Sup, supernatant.

Agonists (e.g., ADP, PAF) stimulating platelet activation commonly promote platelets to mobilize storage Ca^2+^, take up extracellular Ca^2+^, and thereby increase the concentration of cytosolic Ca^2+ ^18. Moreover, platelet P-selectin binds to P-selectin glycoprotein ligand-1(PSG-1) on neutrophils, thus promoting the initial binding of the cells. However, firm platelet-neutrophil adhesion is mediated by integrin αMβ2 binding to platelet GPIb or to fibrinogen. Therefore, we observed the calcium mobilization in platelets stimulated by SLY in initial time. We used confocal laser scanning microscopy and Fluo-8 to monitor the calcium mobilization in platelets. In Hank’s balanced salt solution (HBSS) containing 2 mM Ca^2+^, 1 μg/mL rSLY rapidly increased the intracellular Ca^2+^ of platelets (Fig. 4A and Supplementary Video 1), whereas cholesterol (100 μg/mL) strongly diminished the rSLY-induced Ca^2+^ influx (Fig. 4B and Supplementary Video 2). Moreover, the non-hemolytic recombinant mutant, rSLY^P353V^ (Fig. 4C and Supplementary Video 3) and the PBS control (Supplementary Video 4) did not induce a Ca^2+^ influx. It’s interesting that about ~20% cells were observed with increase of Ca^2+^ in short time after stimulating by rSLY, but not to all (Supplementary Video 1). Therefore, we hypothized that pore formation in some platelets (~28%) leading to calcium influx, which served as a positive feedback mechanism for activation of other platelets and ultimately induction of PNC formation. These results indicate that the rSLY-induced Ca^2+^ influx depend on pore formation induced by rSLY. The roles of Ca^2+^ influx in platelet activation and PNC formation were further studied using EGTA to chelate the extracellular Ca^2+^. Blockage of the Ca^2+^ influx by EGTA abrogated the rSLY-induced P-selectin release (CD62P; Fig. 4D) and the P-selectin-mediated PNC formation (Fig. 4E) in human blood (*P* < 0.05). Taken together, these results indicate that the pore formation-dependent Ca^2+^ influx played essential roles in platelet activation and PNC formation induced by SLY.

### Platelet signalling is involved in SLY-induced PNC formation

**Figure 5:**
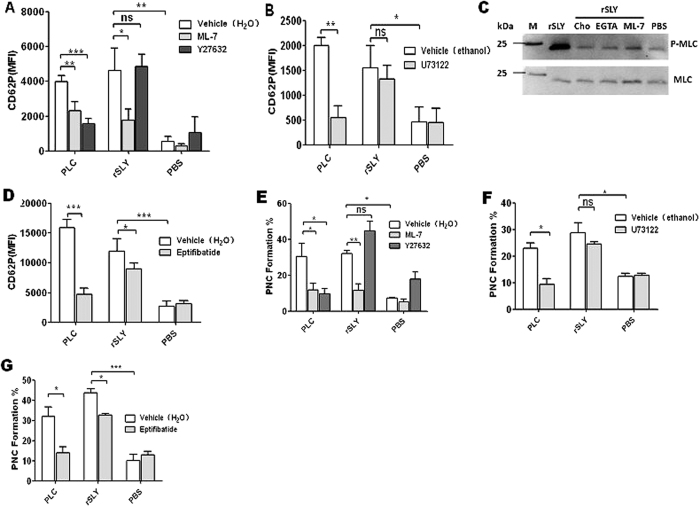
Platelet signalling in response to SLY-induced PNC formation. (**A,B**) The SLY-induced CD62P release in human blood was assessed after incubation with or without MLCK inhibitor ML-7 (100 μM), ROCK inhibitor Y27632 (100 μM), PLC-β inhibitor U73122 (20 μM). *Clostridium perfringens* phospholipase C (PLC) served as a positive control. (C) rSLY-induced myosin light chain (MLC) phosphorylation and the effects of cholesterol/EGTA/ML-7. Human platelets were preincubated with 3 mM EGTA for 10 min or 100 μM ML-7 for 60 min at 37 °C and then stimulated with either 1 μg/mL rSLY for another 10 min. In cholesterol inhibiting assay, rSLY was pretreated by cholesterol (10 μg/mL). After rSLY stimulation, platelets were lysed and electrophoresed. Western blot analysis was performed on platelet lysates using anti-phospho-MLC antibody (1:1000)/anti-MLC antibody (1:1000) and peroxidase-conjugated secondary antibodies, respectively. Similar results were obtained in two separate experiments. (**D**) The SLY-induced CD62P release in human blood was assessed after incubation with or without eptifibatide (10 μM). *Clostridium perfringens* phospholipase C (PLC) served as a positive control. (**E–G**) SLY-induced PNC formation in human blood was assessed after incubation with or without MLCK inhibitor ML-7 (100 μM), ROCK inhibitor Y27632 (100 μM), PLC-β inhibitor U73122 (20 μM) or eptifibatide (10 μM). *Clostridium perfringens* phospholipase C (PLC) served as a positive control. PBS is the negative control for rSLY protein. ML-7, Y27632 and eptifibatide were dissolved in H_2_O. U73122 was dissolved in ethanol. Data in (**A,B,D–G**) are given as the mean ± SD of three independent experiments from three different blood donors. *P < 0.05; **P < 0.01; ns, no significance.

To investigate the possible signalling in platelet activation induced by SLY, specific inhibitors were used. Firstly, *Clostridium perfringens* phospholipase C (PLC) use as positive control to detect the activities of specific inhibitors (Fig. 5). Secondly, the effects of specific inhibitors on SLY-induced PNC formation were detected. Interestingly, the MLCK-specific inhibitor, ML-7, dramatically reduced the SLY-induced P-selectin release (Fig. 5A), however, both the ROCK-specific inhibitor, Y27632, and the PLC-β-specific inhibitor, U73122, did not (Fig. 5A,B). Moreover, rSLY strongly induced myosin light chain (MLC) phosphorylation, while cholesterol, EGTA and ML-7 all had apparent inhibitory effect on MLC phosphorylation induced by rSLY (Fig. 5C). The phosphorylation of MLC usually leads to rearrangement of the platelet cytoskeleton, and then promoting the mobilization of P-selectin from α-granules to the plasma membrane, in which subsequently mediates the platelet–neutrophil interactions^14^. As expected, ML-7 also strongly inhibited the SLY-induced PNC formation, but Y27632 and U73122 did not (Fig. 5E,F). Thus, we propose that the PLC-β-IP3/DAG-MLCK and Rho-ROCK-MLCK pathways are not involved in SLY-induced platelet activation and PNC formation, but that the SLY-induced pore formation dependent-Ca^2+^ influx directly activates the Ca^2+^-calmodulin-MLCK signalling.

Agonists (e.g., ADP, PAF) activate human platelets, resulting in an “inside-out” signalling, leading to integrin GPIIb/IIIa activation that promotes binding to fibrinogen in the plasma. In turn, the fibrinogen-occupied GPIIb/IIIa usually reinforces activation of “outside-in” signalling, leading to secretion of α-granules. In fact, in our early study, SLY induced Fg binding to platelet in human blood (Front. Cell. Infect. Microbiol. 2016, accepted). Eptifibatide is a specific inhibitor of fibrinogen binding to the GPIIb/IIIa receptor. Herein, we used eptifibatide to explore the role of “outside-in” signalling in SLY-induced PNC formation. Pre-incubation of eptifibatide with human blood had some inhibiting effects on P-selectin release (Fig. 5D, *P* < 0.05) and PNC formation (Fig. 5G, *P* < 0.05), suggesting that the “outside-in” signalling was also involved in SLY-induced PNC formation, but with a smaller effect than the Ca^2+^-MLCK signalling.

### Other CDCs that promote PNC formation require pore formation-dependent calcium influx

**Figure 6:**
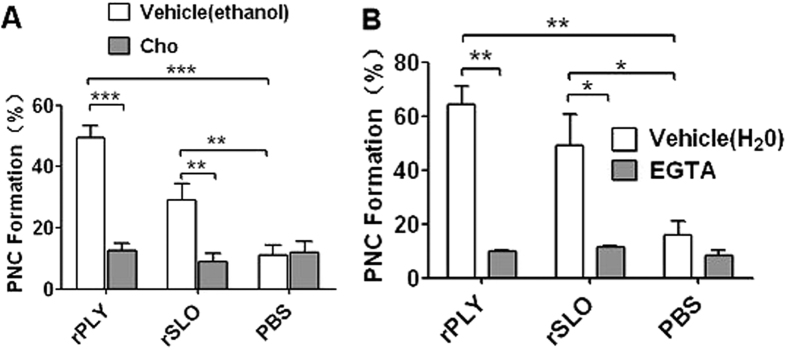
rPLY or rSLO-induced PNC formation via pore formation-dependent Ca^2+^ influx. (**A**) The cholesterol (100 μg/mL) effect on rPLY (0.8 μg/mL) or rSLO (1.5 μg/mL)-induced PNC formation. (**B**) The EGTA (3 mM) effect on rPLY (0.8 μg/mL) or rSLO (1.5 μg/mL)-induced PNC formation. PBS is the negative control for recombinant proteins. Cholesterol and EGTA were dissolved in ethanol and H_2_O, respectively. Data in A and B are given as the mean ± SD of three independent experiments from three different blood donors. *P < 0.05; **P < 0.01; ***P < 0.01; ns, no significance; rSLY, suilysin; Cho, cholesterol; 0.8 + Cho, 0.8 μg/mL of rPLY added to cholesterol; 1.5 + Cho, 1.5 μg/mL of rSLO added to cholesterol.

PLY and SLO are important toxins of *S. pneumoniae* and *S. pyogenes*, respectively, which belong to the Group I typical CDCs with high affinity to cholesterol and high structural similarity to SLY^20^,^21^. Therefore, PLY and SLO possibly share similar functional characteristics to SLY. Interestingly, cholesterol completely abrogated their induction of PNC formation (Fig. 6A). Moreover, EGTA suppressed PNC formation induced by PLY or SLO (Fig. 6B). We propose that CDC-mediated PNC formation is a similar mechanism used by a wide range of bacteria, which is triggered by the pore formation-dependent Ca^2+^ influx.

## Discussion

In the current study, we found that SLY, which was secreted from *S. suis* 05ZYH33 at the stationary growth phase, stimulated PNC formation and that this stimulation required SLY-induced Ca^2+^ influx and P-selectin release. In contrast, *S. suis* 05ZYH33 cells did not induce PNC formation. These results indicate that *S. suis* 05ZYH33 may not directly interact with platelets, instead they cause PNC formation via SLY. 05ZYH33 is a sequenced strain that belongs to the sequence type 7 (ST-7) strains, and caused the 2005 outbreak and STSS in China^25^. Moreover, ST-7 strains were more toxic to human peripheral blood mononuclear cells (PBMCs) than ST-1 strains (mainly referring to the European virulent strains)^26^. In particular, we have previously shown that the ST-7 strains produced more SLY than the non-epidemic strains, contributing to invasive infection^27^. Therefore, SLY may be a potential therapeutic target for preventing *S. suis*-mediated platelet activation, thrombocytopenia, liver failure and purpura gangrenosa.

Similar to our findings, other cytolysins of the CDC family have been found to stimulate platelet activation and PNC formation. Bryant *et al*. have reported that SLO induced coaggregation of platelets and neutrophils, and stimulated platelet P-selectin (CD62P)-dependent PNC formation^9^. Parimon *et al*. have also found that *Staphylococcus aureus* α-hemolysin promotes platelet-neutrophil aggregate formation via P-selectin^10^. However, the molecular mechanism underlying these bacterial toxins-induced P-selectin activation and PNC formation are not fully understood. Although it has been reported that there are 20 kinds of bacterial cytolysin belonging to Group I CDCs with high affinity to cholesterol^28^, there is only little evidence showing associations between Group I CDCs and platelet activation. The current study demonstrated direct evidence showing that SLY induced Ca^2+^ influx in platelets, which was required for SLY-induced PNC formation. Similar to SLY, the other two CDCs, PLY and SLO, also induced PNC formation in a Ca^2+^ influx-dependent manner. These data suggest that CDCs form pores in platelet membranes, which result in Ca^2+^ influx, consequently triggering intracellular signalling events that induce PNC formation.

We also found that SLY-induced P-selectin (CD62P) release was Ca^2+^ influx dependent, suggesting that stimulation of this adhesion factor is downstream of the SLY-induced Ca^2+^ influx. The signalling events between the Ca^2+^ influx and the P-selectin release or PNC formation strongly associated with MLCK activity, because the MLCK inhibitor, ML-7, significantly reduced the SLY-induced platelet P-selectin release and the P-selectin-mediated PNC formation. However, these processes did not involve PLC-β-IP3/DAG-MLCK or Rho-ROCK-MLCK signalling (Fig. 5A,B,D,E); the SLY-induced Ca^2+^ influx may directly bind to calmodulin, which consequently activates MLCK. These data imply that the signalling in response to SLY may be different from that in response to typical platelet agonists such as ADP and thromboxane A2 (TXA2)^14^. Moreover, eptifibatide, a fibrinogen-specific inhibitor that binds to GPIIb/IIIa receptor, showed smaller inhibition effects on PNC formation, which is consistent with Bryant *et al*.^9^. These authors suggested that platelet GPIIb/IIIa also participates in PNC formation using fibrinogen as a bridging molecule between GPIIb/IIIa and PMNL CD11b/CD18. In contrast, we believe that the “outside-in” signalling plays minor roles in PNC formation, because eptifibatide also inhibited the P-selectin release in α-granules.

**Figure 7:**
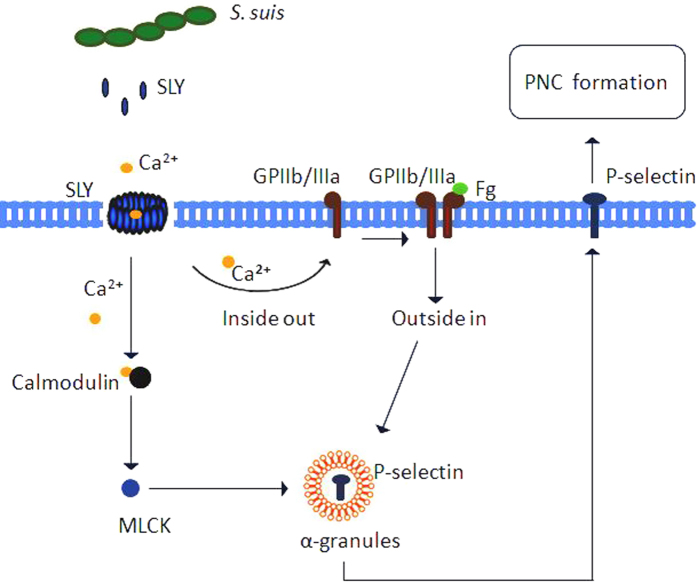
Schematic representation of PNC formation induced by *S. suis*. The Ca^2+^ influx across transmembrane pores created by SLY, the cholesterol-dependent cytolysin (CDC) of *S. suis*, triggers the Ca^2+^-MLCK and the “outside-in” signalling, leading to α-granules (P-selectin) release to the platelets surface. Subsequently, P-selectin mediates the platelet adhesion to neutrophils to form PNC.

In summary (Fig. 7), we found that SLY was secreted by *S. suis* 05ZYH33 at the stationary growth phase. The Ca^2+^ influx across transmembrane pores created by SLY triggered the Ca^2+^-MLCK and the “outside-in” signalling, leading to α-granules (P-selectin) release to the platelets’ surface. Subsequently, P-selectin mediated the platelets adhesion to neutrophils to form PNC. These findings imply that SLY and MLCK may be potential therapeutic targets for preventing *S. suis*-induced platelet activation and related disorders.

## Methods

### Ethics statement

The healthy donors who provide the blood in this study provided written informed consent in accordance with the Declaration of Helsinki. Approval was obtained from the Institutional Medical Ethics Committee of the Academy of Military Medical Sciences. The human blood used in this study were obtained in accordance with the approved guidelines.

### Reagents

Monoclonal mouse anti-human antibodies, including FITC-conjugated anti-CD42b (clone HIP1), APC-conjugated anti-CD11b (clone M1/70), PE-conjugated anti-CD62P (clone AK-4), anti-CD62P (clone AK-4), and isotype control antibody were from BD Bioscience. Mouse anti-phospho-myosin light chain 2 (MLC) antibody (mAb#3675) and rabbit anti-myosin light chain 2 (MLC) antibody (mAb#3672) were from (Cell Signaling Technology). U73122, ML-7, Y27632 and eptifibatide acetate, which are inhibitors of phospholipase C (PLC), myosin light chain kinase (MLCK), rho-associated, coiled-coil-containing protein kinase (ROCK) and CD41a, respectively, were from Sigma-Aldrich. *Clostridium perfringens* phospholipase C (PLC), cholesterol and EGTA were also purchased from Sigma-Aldrich. Quest Fluo-8 calcium fluorescence probe was from AAT Bioquest^®^, Inc. Wright’s stain was from Beijing CellChip Biotechnology Co., Ltd.

### Strains and culture supernatant

*S. suis* 05ZYH33 was originally isolated from an STSS patient^29^. The isogenic *sly* mutant Δsly was constructed in our previous study^27^. The Canadian avirulent strain 1330, which does not express SLY, was donated by Prof Marcelo Gottschalk (Université de Montréal). Group A streptococcus (GAS) was M1 type E477. *S. suis* strains were cultured in Todd-Hewitt broth (THB, BD Biosciences) at 37 °C for 4 h (OD_600_ = 0.4, exponential growth phase) or 6 h (OD_600_ = 1.0, stationary growth phase), and then harvested for experiments. *S. suis* strains and GAS cells were washed three times with PBS, and then re-suspended in PBS at a density of 2 × 10^9^ CFU/mL to use for stimulation.

### Preparation of recombinant suilysin (rSLY), pneumolysin (rPLY), streptolysin O (rSLO) and factor H-binding protein (rFhb)

rSLY, rPLY, rSLO and (rFhb) used in this study were prepared as previously reported^29^,^30^.

### Human platelets

Whole blood of healthy donors, who did not use anti-platelet drugs within the previous 15 days, was drawn in the 307 Hospital (China), and the blood was collected in tubes containing sodium citrate (the final concentration was 3.2%)^31^. Each donor signed an informed consent form. The procedure of collecting and handling the blood samples was in accordance with the Declaration of Helsinki and was approved by the Institutional Review Board of the 307 Hospital. A total of 6 mL of whole blood was centrifuged at 900 rpm for 10 min in a horizontal centrifuge (Sigma-Aldrich), to prepare platelet-rich plasma (PRP 3 × 10^8^/mL).

### PNC formation

Flow cytometry was performed to analyse PNC formation^10^. Briefly, whole blood (100 μL) was mixed with 15 μL of FITC-conjugated anti-human CD42b and APC-conjugated anti-human CD11b, and incubated at 37 °C for 10 min. The blood was then stimulated with 10 μL of SLY, supernatant, THB, PBS or other regents at 37 °C for 10 min, and then the blood was immediately prepared for flow cytometry using a commercial formic-acid red cell lysis/formalin cell-fixation kit (Coulter^®^ Q-prep) according to the manufacturer’s instructions. Flow cytometry was performed on an Accuri C6 flow cytometer (BD Biosciences). The granulocyte gate was drawn based on the forward- and side-scatter profiles of the sample. CD11b-positive events within this gate were analysed to estimate the proportion of CD42b-positive platelets. The above samples were then visualised using an FV1000 confocal laser scanning microscope (Olympus) by XY scanning. In CD62P blocking assay, human blood was pre-incubated with anti-CD62P antibody (10 μg/mL) or the isotype IgG (10 μg/mL) at 37 °C for 10 min before being exposed to rSLY or supernatant stimulus.

### Observation of PNC formation in whole blood by microscopy

Whole blood (100 μL) was incubated with 10μL of SLY (1 μg/mL) or other stimuli at 37 °C for 10 min, and then 5 μL of the blood sample were used to prepare a blood smear on a glass slide. After the blood smear dried completely, Wright’s stain was added to cover the blood smear and an equal volume of PBS (pH 6.4–6.8) was added 1 min later. The Wright’s stain and PBS were gently mixed and the blood smear was incubated with the mixture for 5 min. The slide was then washed gently with distilled water and observed under an oil-immersion objective (Olympus).

### Flow cytometry analysis of CD62P

Flow cytometry was performed to analyse the expression of P-selectin (CD62P) as previously described^10^. Briefly, 100 μL of heparinized human blood were incubated with 15 μL of PE-conjugated anti-human CD62P antibody at 37 °C for 10 min. Then, rSLY or other stimuli was added and the blood was incubated for another 15 min. One millilitre of red cell lysis buffer containing formalin was then added to the blood to lyse red cells and to fix the platelets at room temperature for 8 min. After the red blood cells were lysed completely, the blood was centrifuged and the cell pellet was washed with PBS. The cell pellet was re-suspended in 300 μL of PBS and analysed with an Accuri C6 flow cytometer (BD Biosciences). The expression of CD62P was determined by mean fluorescence intensity (MFI) of 10000 events in each sample. To test the effect of cholesterol, SLY was pre-incubated with cholesterol (100 μg/mL) for 10 min, and then blood was incubated with the mixture. To test the effect of EGTA, blood was pre-incubated with EGTA (3 mM) for 10 min, and then incubated with rSLY or other stimuli. Representative histograms of the MFI of CD62P are displayed. Data are expressed as mean MFI ± SD of three independent experiments, with each experiment using blood from a different donor.

### Flow cytometry analysis of SLY binding

To analyse the binding of rSLY/rSLY^P353V^ to platelets, firstly, rSLY/rSLY^P353V^ or the control BSA were marked by Alexa Fluor^®^647, which was performed as described in the generic protocol Amine-Reactive Probes (MP00143). Secondly, the Alexa Fluor^®^647-rSLY/rSLY^P353V^ or BSA (1 μg/mL) were incubated with platelets at RT for 10 min, and then washed twice with PBS. The binding of rSLY/rSLY^P353V^ to platelets was analysed by Accuri C6 flow cytometer (BD Biosciences). The protein binding was determined by mean fluorescence intensity (MFI) of 10000 events in each sample. Data are expressed as mean MFI ± SD of three independent experiments, with each experiment using blood from a different donor. In cholesterol inhibiting assay, rSLY was pretreated by cholesterol (100 μg/mL).

### Calcium influx in platelets

A total of 200 μL of PRP was centrifuged at 2000 rpm for 10 min, and then washed twice with PBS. Subsequently, 100 μL of the calcium probe, Fluo-8 (5 μM), were added and incubated at 37 °C for 30 min. The PRP was centrifuged at 2000 rpm for 10 min, and then washed twice with 200 μL of PBS to remove free calcium probe. The PRP was re-suspended in 180 μL of HBSS (containing 2 mM Ca^2+^) or D-HBSS (without Ca^2+^), and then 20 μL of SLY (1 μg/mL), rSLY^P353V^ (1 μg/mL) or cholesterol (100 μg/mL) were added. Ca^2+^ influx in a Petri dish was measured by an FV1000 confocal laser scanning microscope (Olympus) by XYT scanning.

### Immunoblotting to detect the phosphorylation of MLC

Whole platelet lysates from the same cell equivalents were electrophoresed on a SDS-polyacrylamide gel and transferred onto nitrocellulose membranes. The membranes were blocked overnight in 5% BSA in Tris-buffered saline supplemented with 0.1% Tween 20 (TBST), washed three times in TBST, and the membrane was immunoblotted with primary antibodies against MLC (1:1000) or phospho-MLC (1:1000). After washing with TBST, the membrane was incubated with horseradish peroxidase-conjugated secondary antibodies for 1 h. After washing with TBST, protein bands on the membrane were detected using ECL Western blotting detection reagents (Thermo Scientific) and visualized by molecular imager (ChemiDOC^TM^ XRS+ with Image Lab^TM^ Software).

### Statistical analysis

Unless otherwise specified, all the data are expressed as mean ± SD. All PNC assays were performed on human blood from three individual donors in independent experiments. Differences between two groups were analysed using the unpaired two-tailed Student’s *t*-test, if the Levene’s test was not significant (*P* > 0.05), otherwise, Mann–Whitney tests were used. For all tests, *P* < 0.05 was considered statistically significant. All statistical analyses were carried out using SPSS 15.0 (SPSS Inc.).

## Additional Information

**How to cite this article**: Zhang, S. *et al*. Suilysin-induced Platelet-Neutrophil Complexes Formation is Triggered by Pore Formation-dependent Calcium Influx. *Sci. Rep*. **6**, 36787; doi: 10.1038/srep36787 (2016).

**Publisher’s note:** Springer Nature remains neutral with regard to jurisdictional claims in published maps and institutional affiliations.

## Supplementary Material

Supplementary Information

Supplementary Video S1

Supplementary Video S2

Supplementary Video S3

Supplementary Video S4

## References

[b1] Wertheim H. F. L., Nghia H. D. T., Taylor W. & Schultsz C. Streptococcus suis: An Emerging Human Pathogen. Clin Infect Dis. 48, 617–625 (2009). 19191650 10.1086/596763

[b2] Huong V. T. . Epidemiology, clinical manifestations, and outcomes of Streptococcus suis infection in humans. Emerg Infect Dis. 20, 1105–1114 (2014). 24959701 10.3201/eid2007.131594 PMC4073838

[b3] Sriskandan S. & Slater J. D. Invasive disease and toxic shock due to zoonotic Streptococcus suis: an emerging infection in the East? PLoS Med 3, e187 (2006). 16594733 10.1371/journal.pmed.0030187 PMC1434506

[b4] Tang J. . Streptococcal toxic shock syndrome caused by Streptococcus suis serotype 2. Plos Med 3, 668–676 (2006). 10.1371/journal.pmed.0030151 PMC1434494 16584289

[b5] Yu H. J. . Human Streptococcus suis outbreak, Sichuan, China. Emerg Infect Dis 12, 914–920 (2006). 16707046 10.3201/eid1206.051194 PMC3373052

[b6] Stahl A. L. . Lipopolysaccharide from enterohemorrhagic Escherichia coli binds to platelets through TLR4 and CD62 and is detected on circulating platelets in patients with hemolytic uremic syndrome. Blood 108, 167–176 (2006). 16514062 10.1182/blood-2005-08-3219 PMC1895830

[b7] Peters M. J. . Severe meningococcal disease is characterized by early neutrophil but not platelet activation and increased formation and consumption of platelet-neutrophil complexes. J Leukoc Biol 73, 722–730 (2003). 12773504 10.1189/jlb.1002509

[b8] Bryant A. E. . Clostridial gas gangrene. II. Phospholipase C-induced activation of platelet gpIIbIIIa mediates vascular occlusion and myonecrosis in Clostridium perfringens gas gangrene. J Infect Dis 182, 808–815 (2000). 10950775 10.1086/315757

[b9] Bryant A. E. . Vascular dysfunction and ischemic destruction of tissue in Streptococcus pyogenes infection: the role of streptolysin O-induced platelet/neutrophil complexes. J Infect Dis 192, 1014–1022 (2005). 16107954 10.1086/432729

[b10] Parimon T. . Staphylococcus aureus alpha-hemolysin promotes platelet-neutrophil aggregate formation. J Infect Dis 208, 761–770 (2013). 23698812 10.1093/infdis/jit235 PMC3733505

[b11] Evangelista V. . Platelet/polymorphonuclear leukocyte interaction: P-selectin triggers protein-tyrosine phosphorylation-dependent CD11b/CD18 adhesion: role of PSGL-1 as a signaling molecule. Blood 93, 876–885 (1999). 9920836

[b12] McEver R. P. Selectins. Curr Opin Immunol 6, 75–84 (1994). 7513527 10.1016/0952-7915(94)90037-x

[b13] Yang J., Furie B. C. & Furie B. The biology of P-selectin glycoprotein ligand-1: its role as a selectin counterreceptor in leukocyte-endothelial and leukocyte-platelet interaction. Thromb Haemost 81, 1–7 (1999). 10348699

[b14] Zarbock A., Polanowska-Grabowska R. K. & Ley K. Platelet-neutrophil-interactions: linking hemostasis and inflammation. Blood Rev 21, 99–111 (2007). 16987572 10.1016/j.blre.2006.06.001

[b15] Rebecchi M. J. & Pentyala S. N. Structure, function, and control of phosphoinositide-specific phospholipase C. Physiol Rev 80, 1291–1335 (2000). 11015615 10.1152/physrev.2000.80.4.1291

[b16] Rhee S. G. Regulation of phosphoinositide-specific phospholipase C. Annu Rev Biochem 70, 281–312 (2001). 11395409 10.1146/annurev.biochem.70.1.281 PMC4781088

[b17] Klages B., Brandt U., Simon M. I., Schultz G. & Offermanns S. Activation of G12/G13 results in shape change and Rho/Rho-kinase-mediated myosin light chain phosphorylation in mouse platelets. J Cell Biol 144, 745–754 (1999). 10037795 10.1083/jcb.144.4.745 PMC2132941

[b18] Bird G. S. . Mechanisms of phospholipase C-regulated calcium entry. Curr Mol Med 4, 291–301 (2004). 15101686 10.2174/1566524043360681

[b19] Bennett J. S. Structure and function of the platelet integrin alphaIIbbeta3. J Clin Invest 115, 3363–3369 (2005). 16322781 10.1172/JCI26989 PMC1297263

[b20] Giddings K. S., Zhao J., Sims P. J. & Tweten R. K. Human CD59 is a receptor for the cholesterol-dependent cytolysin intermedilysin. Nat Struct Mol Biol 11, 1173–1178 (2004). 15543155 10.1038/nsmb862

[b21] Xu L. . Crystal structure of cytotoxin protein suilysin from Streptococcus suis. Protein Cell 1, 96–105 (2010). 21204001 10.1007/s13238-010-0012-3 PMC4875105

[b22] Gottschalk M. G., Lacouture S. & Dubreuil J. D. Characterization of Streptococcus suis capsular type 2 haemolysin. Microbiology 141 (Pt 1), 189–195 (1995). 7894711 10.1099/00221287-141-1-189

[b23] Rossjohn J., Feil S. C., McKinstry W. J., Tweten R. K. & Parker M. W. Structure of a cholesterol-binding, thiol-activated cytolysin and a model of its membrane form. Cell 89, 685–692 (1997). 9182756 10.1016/s0092-8674(00)80251-2

[b24] Gilbert R. J. . Two structural transitions in membrane pore formation by pneumolysin, the pore-forming toxin of Streptococcus pneumoniae. Cell 97, 647–655 (1999). 10367893 10.1016/s0092-8674(00)80775-8

[b25] Ye C. . Clinical, experimental, and genomic differences between intermediately pathogenic, highly pathogenic, and epidemic Streptococcus suis. J Infect Dis 199, 97–107 (2009). 19016627 10.1086/594370

[b26] Ye C. . Streptococcus suis sequence type 7 outbreak, Sichuan, China. Emerg Infect Dis 12, 1203–1208 (2006). 16965698 10.3201/eid1208.060232 PMC3291228

[b27] He Z. . Increased production of suilysin contributes to invasive infection of the Streptococcus suis strain 05ZYH33. Mol Med Rep 10, 2819–2826 (2014). 25241621 10.3892/mmr.2014.2586 PMC4227431

[b28] Tabata A. . The diversity of receptor recognition in cholesterol-dependent cytolysins. Microbiol Immunol 58, 155–171 (2014). 24401114 10.1111/1348-0421.12131

[b29] Pian Y. . Fhb, a novel factor H-binding surface protein, contributes to the antiphagocytic ability and virulence of Streptococcus suis. Infect Immun 80, 2402–2413 (2012). 22526676 10.1128/IAI.06294-11 PMC3416472

[b30] Ren Z. Q. . Construction and activities of suilysin mutants. Xi Bao Yu Fen Zi Mian Yi Xue Za Zhi 28, 580–582 (2012). 22691347

[b31] Shannon O. . Severe streptococcal infection is associated with M protein-induced platelet activation and thrombus formation. Mol Microbiol 65, 1147–1157 (2007). 17662041 10.1111/j.1365-2958.2007.05841.x

