# Supplementary material for: Suilysin-induced Platelet-Neutrophil Complexes Formation is Triggered by Pore Formation-dependent Calcium Influx
Source: Sci Rep. 2016 Nov 10;6:36787. doi: 10.1038/srep36787 (PMC5103290; doi:10.1038/srep36787)
Supplement: Supplementary Information [file srep36787-s1.pdf]

Suilysin-induced Platelet-Neutrophil Complexes Formation is Triggered by Pore Formation-dependent Calcium Influx

Shengwei Zhang[1,2], Yuling Zheng[1], Shaolong Chen[1], Shujing Huang[3], Keke Liu[1], Qingyu Lv[1], Yongqiang Jiang[1*], Yuan Yuan[1*]

[1] *State key Laboratory of Pathogen and Biosecurity, Beijing Institute of Microbiology and Epidemiology, Beijing 100071, China;*

[2] *Department of clinical laboratory, Dongfang Hospital, Beijing University of Chinese Medicine, Beijing 100078, China*

[3] *Department of clinical laboratory, Beijing You'an hospital, Capital Medical University, Beijing 100069, China*

[#]S.Z. and Y.Z. contributed equally to this article as first author.

*Corresponding author: Dr. Yuan Yuan and Pro. Yongqiang Jiang

State Key Laboratory of Pathogen and Biosecurity, Beijing Institute of Microbiology and Epidemiology, Beijing 100071

Tel : (86-10) 66948487. Fax: (86-10) 66948637.

E-mail: miniminiyuan@163.com (Y.Y.) or jiangyq@nic.bmi.ac.cn (Y.J.)

Key Words: *Streptococcus suis*; Suilysin; Platelet-Neutrophil Complexes; Calcium influx

Running Title: Suilysin-induced Platelet-Neutrophil Complexes Formation

Video 1. The rSLY induced-Ca^{2+} influx in human platelets. The purified platelets marked with Fluo-8 were resuspended in HBSS (with 2 mM Ca^{2+}) and stimulated by rSLY (1 g/mL). The Ca^{2+} influx in platelets was observed using an FV1000 confocal laser scanning microscope.

Video 2. The effect of cholesterol on rSLY induced-Ca^{2+} influx in human platelets. The purified platelets marked with Fluo-8 were resuspended in HBSS (with 2 mM Ca^{2+}) and stimulated by rSLY (1 g/mL) that was pretreated by cholesterol (10 g/mL). The Ca^{2+} influx in platelets was observed using an FV1000 confocal laser scanning microscope.

Video 3. The $rSLY^{P353V}$ induced-Ca^{2+} influx in human platelets. The purified platelets marked with Fluo-8 were resuspended in HBSS (with 2 mM Ca^{2+}) and stimulated by $rSLY^{P353V}$ (1 g/mL). The Ca2+ influx in platelets was observed using an FV1000 confocal laser scanning microscope.

Video 4. The effect of PBS control on rSLY induced-Ca^{2+} influx in human platelets. The purified platelets marked with Fluo-8 were resuspended in HBSS (with 2 mM Ca^{2+}) and stimulated by PBS control. The Ca2+ influx in platelets was observed using an FV1000 confocal laser scanning microscope.
